# Leaf Nutrient Resorption Efficiency Aligns with the Leaf but Not Root Economic Spectrum in a Tropical Mangrove Forest

**DOI:** 10.3390/plants14172610

**Published:** 2025-08-22

**Authors:** Dalong Jiang, Tao Nie, Qiuyu He, Zuo Xu, Han Y. H. Chen, Erhui Feng, Josep Peñuelas

**Affiliations:** 1 Ministry of Education Key Laboratory for Ecology of Tropical Islands, Key Laboratory of Tropical Animal and Plant Ecology of Hainan Province, College of Life Sciences, Hainan Normal University, Haikou 571158, China; 202313095400004@hainnu.edu.cn (T.N.); hqy6965@163.com (Q.H.); xzuo1243@hainnu.edu.cn (Z.X.); 2 Hainan Dongzhaigang Mangrove Ecosystem Provincial Observation and Research Station, Haikou 571129, China; 3 Faculty of Natural Resources Management, Lakehead University, 955 Oliver Road, Thunder Bay, ON P7B 5E1, Canada; han.yh.chen@gmail.com; 4 Institute for Global Change Biology, School for Environment and Sustainability, University of Michigan, 440 Church Street, Ann Arbor, MI 48109, USA; 5 Hainan Dongzhaigang National Nature Reserve Authority, Haikou 571129, China; onlyonefengerhui@163.com; 6 CSIC, Global Ecology Unit CREAF-CSIC-UAB, Bellaterra, 08193 Barcelona, Catalonia, Spain; 7 CREAF, Cerdanyola del Vallès, 08193 Barcelona, Catalonia, Spain

**Keywords:** N and P resorption efficiency, shrub and tree, green leaf nutrients, fine root, leaf structural traits, mangrove

## Abstract

Leaf nutrient resorption efficiency (NuRE) is critical for plant nutrient conservation, yet its relationship with leaf and root economic traits remains poorly understood in mangroves. We quantified nitrogen (N) and phosphorus (P) resorption across ten mangrove species (five trees and five shrubs) in Hainan, China, and related NuRE to key leaf (leaf mass per area, LMA; leaf dry mass content, LDMC; and green leaf nitrogen and phosphorus contents, N_gr_ and P_gr_, respectively) and root (specific root length, SRL; root tissue density, RTD; root diameter, RD; and root nitrogen content, N_root_) traits. We found that species with a lower leaf structural investment (LMA = 103–173 g m^−2^, LDMC = 19–27%) presented a 6–45% greater N and P resorption efficiency than those with a higher structural investment (LMA = 213–219 g m^−2^, LDMC = 26–31%). Contrary to global meta-analyses, higher green leaf N and P contents also predicted a greater NuRE, implying enhanced internal recycling under chronic nutrient limitation. Root traits (SRL, RTD, RD, and N_root_) had no significant influence on NuRE, indicating decoupled above- versus belowground strategies. Trees and shrubs diverged in size but converged in NuRE–leaf trait relationships. These findings refine plant economics theory and guide restoration by prioritizing species with acquisitive, high-NuRE foliage for nutrient-poor coasts.

## 1. Introduction

Leaf nutrient resorption is a critical physiological process in which plants remobilize nutrients from senescing leaves before they abscise [1,2,3]. Nutrient resorption efficiency (NuRE), the proportion of nutrients resorbed from senescing leaves, serves as a key indicator of both plant nutrient limitation and ecosystem resilience, directly linking organismal physiology to biogeochemical cycles [4]. This strategy not only conserves resources but also plays a significant role in supporting plant growth and reproduction [5]. By recycling these nutrients, plants can meet more than 30% of their annual nutrient demands, which is essential for their overall health and fitness [6,7]. This mechanism reduces the dependence of plants on soil nutrients, highlighting their ability to thrive in diverse environmental conditions [8,9]. Moreover, leaf nutrient resorption is a key driver of plant nutrient economy and litter quality, thereby significantly influencing nutrient cycling within ecosystems [9,10]. Despite extensive research on nutrient resorption over the past several decades [11,12,13,14], the relationships between nutrient resorption and plant functional traits remain unclear.

The relationship between NuRE and the leaf economic spectrum (LES) offers critical insights into plant nutrient strategies while revealing persistent discrepancies between theoretical predictions and observations [7,15,16,17]. Ecological theory predicts that fast-growing, acquisitive species, characterized by higher foliar nutrient concentrations [18], exhibit a lower NuRE due to reduced selective pressure for nutrient conservation [19]. Conversely, slow-growing, conservative species are thought to maximize nutrient retention through an increased NuRE, which is supported by traits such as extended leaf longevity [20]. However, recent studies have shown that conservative species often present lower NuRE values [5,21,22], suggesting that alternative nutrient retention strategies may reduce their reliance on resorption. These findings imply that NuRE is regulated independently of structural leaf traits, although the underlying mechanisms remain unclear.

Although the ‘fast–slow’ plant economics spectrum has been extensively characterized across various plant organs [18,23,24], the functional coordination between root nutrient acquisition and leaf nutrient resorption remains largely unresolved. It is hypothesized that root traits influence leaf NuRE through three interconnected mechanisms. First, plants face resource allocation trade-offs: roots with greater structural investment, such as a higher tissue density (RTD), generally show a reduced nutrient uptake capacity, which may select for increased reliance on nutrient resorption from senescing leaves to meet metabolic demands [10]. Second, root foraging strategies directly affect nutrient acquisition pathways; for instance, acquisitive root traits, such as a high specific root length (SRL), elevated nitrogen content in roots (N_root_), and extensive mycorrhizal colonization, increase soil nutrient capture [25,26,27], potentially reducing the selective pressure for efficient foliar resorption. Conversely, conservative root traits, such as a high RTD, often co-occur with nutrient retention strategies that favor a high NuRE [28]. Third, environmental conditions modulate these relationships: in nutrient-rich environments, plants may prioritize root foraging over resorption efficiency, whereas nutrient-poor conditions favor the development of conservative traits in both roots (high tissue density) and leaves (high NuRE) [29]. These integrated adjustments reflect whole-plant nutrient optimization, highlighting the need to better understand root–leaf coordination in ecosystem nutrient cycling.

Owing to their intertidal habitat, mangroves experience severe nutrient limitations driven by tidal fluctuations, high salinity, and waterlogged soils—conditions that pose significant challenges for nutrient acquisition [30]. These environmental stresses provoked the evolution of efficient nutrient resorption mechanisms; for example, *Kandelia obovata* presented a nitrogen resorption efficiency (NRE) of 70.86% and a phosphorus resorption efficiency (PRE) of 52.73% [14,31]. These traits make mangroves an ideal model for studying how plants adapt to nutrient scarcity. Furthermore, mangrove species display considerable variation in traits both within and among species [32,33]. This diversity provides valuable opportunities for comparative studies to elucidate the specific strategies and adaptations employed by different mangrove species to optimize nutrient acquisition and conservation.

In this study, we investigated NRE and PRE across ten mangrove species (five trees and five shrubs) in northeastern Hainan, China. We measured key leaf economic traits (green leaf N content [N_gr_], green leaf P content [P_gr_], leaf mass per area [LMA], and leaf dry matter content [LDMC]), size-related traits (leaf area [LA] and plant height), and root economic traits (root diameter [RD], SRL, N_root_, and RTD) to assess the interspecific relationships between NuRE and leaf and root resource economics within mangrove ecosystems.

We hypothesized the following:

1. Increased leaf nutrient contents (N_gr_ and P_gr_) reduce NRE and PRE due to decreased selective pressure for internal nutrient conservation, as nutrient-rich species may rely more on soil nutrient uptake than on internal recycling.
2. Species with higher LMA and LDMC are expected to exhibit higher NRE and PRE values, given that these traits are associated with conservative resource-use strategies and an extended leaf lifespan, which promote efficient nutrient retention.
3. Species with higher SRL, N_root_, and RD are likely to have lower NRE and PRE, as these traits indicate an acquisitive strategy focused on nutrient acquisition from the soil rather than conservation through leaf resorption.

## 2. Materials and Methods

### 2.1. Study Site

The study site is located within the Dongzhaigang National Nature Reserve in northeastern Hainan Province, China (110°32′–110°37′ E and 19°51′–20°01′ N). As the first mangrove reserve established in China, the reserve spans an area of 3337.6 ha. The reserve features a semienclosed estuary with a muddy substrate, is fed by four small rivers, and is subject to semidiurnal tidal cycles with an average tidal range of 1.6 to 1.8 m. The climate is characterized as tropical maritime monsoon, with a rainy season from May to October, followed by a dry season from November to April. The average annual rainfall is 1676.4 mm, and the mean annual temperature ranges from 23.3 to 23.8° C [34]. Deforestation ceased in 1986 upon the designation of the bay as a national nature reserve [35]. The reserve supports diverse mangrove flora, with a total of 35 species documented across 25 genera and 18 families. These include 24 species of true mangroves, which are affiliated with 14 genera and 10 families, and 11 species of minor mangroves, which are distributed across 11 genera and 8 families [36]. The dominant mangrove species are *Avicennia marina*, *Aegiceras corniculatum*, *Bruguiera sexangula*, *Ceriops tagal*, and *Rhizophora stylosa*.

### 2.2. Leaf and Fine Root Sampling

For this study, we selected five tree species and five shrub species, informed by prior field investigations and a comprehensive review of the literature [20,37,38]. Four plots (10 m × 10 m) were established for each species, with a minimum distance of 1 km between plots to ensure spatial independence and capture representative conditions across the study area [39]. Given the zonation pattern of mangrove species along the intertidal gradient and the relatively low environmental heterogeneity within each species’ habitat [40,41], this sampling design was deemed appropriate for capturing the key growth characteristics of each species. We implemented a stratified random sampling approach to ensure representativeness while minimizing bias in our data collection. Within each plot, the height (m) of all individuals was recorded to assess species-specific growth patterns. At the peak of the rainy season, we collected 30 current-season, fully expanded, light-exposed mature green leaves (10 leaves × 3 randomly selected adult individuals) from standardized mid-canopy positions (1–4 m height) across all cardinal directions, excluding leaves with >5% herbivory damage or visible pathogen infection. While this mid-canopy sampling method involves standardized light exposure for comparability, it may partially underrepresent shade-adapted foliage. These leaves were then pooled to form a composite sample, thereby enhancing the representativeness of the data collected.

During the dry season, we procured senesced leaves from identical individuals whose green leaves had been sampled during the rainy season. The acquisition of senesced leaves was facilitated by gentle flicking of the branches, a method necessitating the development of an abscission layer at the petiole base, as previously described by Zhang et al. (2015) [12]. This collection was meticulously timed to coincide with the specific senescence period of each species, taking into account the intrinsic variability in leaf senescence patterns across different species. However, this natural variation meant that our sampling window might have excluded leaves that abscised either earlier or later than the peak senescence period for each species. After collection, all leaves were carefully placed in plastic bags and promptly stored in a cooler containing ice to preserve their integrity. The samples were subsequently transported to the laboratory for a comprehensive analysis of leaf traits.

During the rainy season, we conducted root sampling from three individuals per plot, tracing the coarse roots from the stem to the point where fine roots were reached, following established protocols [42]. In accordance with the methods of Sun et al. (2021) [43], we selected five intact fine-root strands of the first three orders from the three individuals within each plot. To ensure sample integrity, we defined intact fine-root strands as those without visible damage, decay, or breaks and with a minimum length of 10 cm [44]. We further applied a diameter threshold of ≤2 mm to maintain consistency and focused on the fine-root segments most active in nutrient uptake. This approach prioritizes root order—a more reliable functional indicator than diameter alone—to distinguish absorptive fine roots (orders 1–3) from transport roots (higher orders) [45]. After extraction, the fine roots were carefully transported to the laboratory on ice packs to preserve their physiological state and stored at 4 °C to maintain optimal conditions for subsequent analysis.

### 2.3. Leaf Structural Traits

The leaf area (LA, cm^2^) was determined using a leaf area meter (LI-3000c, Lincoln, NE, USA). To prepare the leaves for subsequent measurements, we submerged fresh leaves in distilled water to rehydrate them until their water potential approached zero (higher than −0.2 MPa). Once rehydrated, the leaves were gently blotted dry with a paper towel to remove excess water from the leaf blade surface. We then determined the leaf saturated mass (LSM, g) by weighing it on a balance (0.0001 g, Meilen, Meifu Electronics Co., Ltd., Shenzhen, China). These fresh leaves were oven-dried at 65 °C for at least 48 h to determine their dry mass (LDM, g). Finally, the LMA (g m^−2^) was calculated by dividing the LDM by the LA. The leaf dry matter content (LDMC, %) was calculated as the ratio of the LDM to LSM.

### 2.4. Leaf Nutrient Contents and Resorption Efficiency

The collected green and senesced leaves were subjected to oven drying at 65 °C for a minimum of 48 h. The leaves were finely ground into a uniform powder using a crusher, which facilitated the extraction of leaf nutrients for further analysis. To determine the N concentrations in green leaves (N_gr_, g kg^−1^) and senesced leaves (N_sen_, g kg^−1^), we employed a C–N elemental analyzer (Vario MAX CN, Elementar Analysensysteme GmbH, Hanau, Germany). P concentrations in green leaves (P_gr_, g kg^−1^) and senesced leaves (P_sen_, g kg^−1^) were determined via an inductively coupled plasma mass spectrometer (Optima 5300 DV; Perkin Elmer, Waltham, MA, USA).

To mitigate potential underestimations of NuRE, it was calculated using the following formula, which accounts for mass loss during leaf senescence [1,2]:

$$NuRE=\left(1-\frac{{Nu}_{sen}}{{Nu}_{gr}}MLCF\right)\times 100\%$$

where Nu_sen_ and Nu_gr_ represent the nutrient concentrations (g kg^−1^) in senesced and green leaves, respectively. MLCF is the mass loss correction factor, specifically the ratio of the dry mass of senesced leaves to that of green leaves.

### 2.5. Root Economic Traits

Freshly sampled roots were scanned with an Epson V850 scanner (Epson America, Inc., Los Alamitos, CA, USA) and analyzed using WinRHIZO Pro 2019 software (Regents Instruments Inc., Quebec City, QC, Canada) to determine key morphological parameters, including the mean diameter (RD, mm) and total root length (m). Fine roots were then oven dried at 65 °C and weighed to obtain their dry weight (g). Specific root length (SRL, m g^−1^) was calculated by dividing the total root length by its dry weight. Root tissue density (RTD, g cm^−3^) was determined by dividing the root dry mass by its volume, assuming that the root was a cylinder. The oven-dried samples of fine roots were ground to a fine powder for root N content (N_root_, g kg^−1^) analyses.

### 2.6. Statistical Analyses

**Figure A1:**
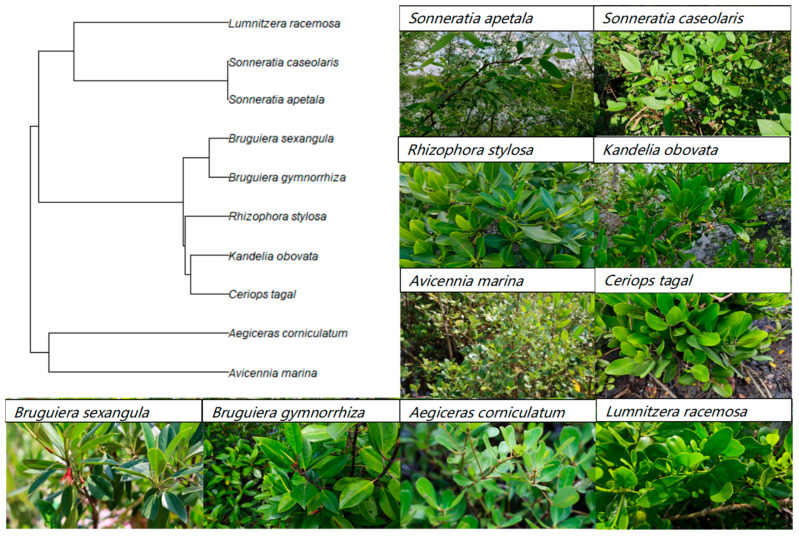
Phylogenetic tree and images of the 10 woody species included in this study.

A phylogenetic tree encompassing all 10 mangrove species was constructed using the ‘V. PhyloMaker’ package in R (Figure A1) [46]. Blomberg’s K and Pagel’s λ, which were calculated with the package ‘phytools’, were used to evaluate the strength of the phylogenetic signal for each plant trait [47,48]. We explored the relationships between leaf NuRE (NRE and PRE) and several leaf economic traits (LMA, LDMC, N_gr_, and P_gr_), size-related traits (LA and height), and root economic traits (SRL, RD, N_root_, and RTD) using Pearson’s correlation analysis. Additionally, simple regression analyses were conducted to scrutinize the influences of leaf economic traits, size-related traits, and root economic traits on NRE and PRE. To investigate the multivariate relationships among these traits, we performed principal component analysis (PCA) of all leaf traits (NRE, PRE, LMA, LDMC, N_gr_, and P_gr_) and size-related traits (LA and height) across all species and within each growth form (shrub and tree) with the ‘vegan’ package. We used permutational multivariate analysis of variance (PERMANOVA) to identify significant differences in these leaf traits between shrubs and trees [27]. All the statistical analyses were performed using R (version 4.3.0), with statistical significance set at *p* < 0.05.

## 3. Results

### 3.1. Trait Variation Across Mangrove Species

Leaf NuRE (NRE and PRE), size-related traits (LA and height), leaf economic traits (N_gr_, P_gr_, LMA, and LDMC), and root economic traits (SRL, RD, N_root_, and RTD) significantly varied among the mangrove species (Table 1 and Table A1). Phylogenetic signal tests indicated a lack of phylogenetic conservatism for all the traits, with values of K and λ less than one and corresponding *p* values greater than 0.05 (Table A2).

### 3.2. Leaf NuRE Links to Economic Traits and Resource-Use Strategies

**Figure 1:**
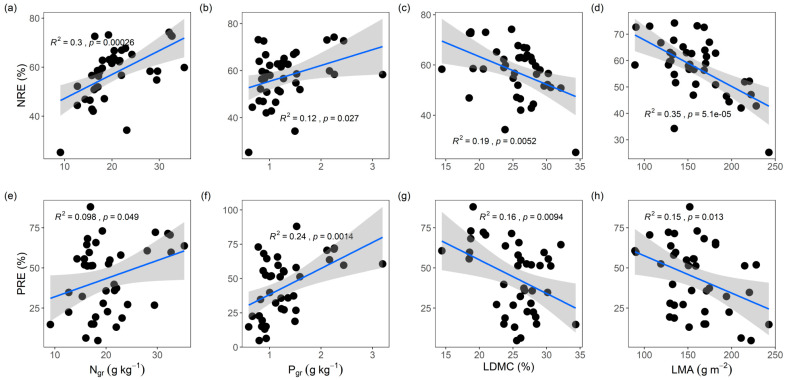
(**a**–**h**) Relationships between leaf nitrogen and phosphorus resorption efficiencies and leaf economic traits. Shaded areas indicate confidence intervals. The *R*^2^ and *p* values are reported. Trait abbreviations are the same as those in Table 1.

**Figure A2:**
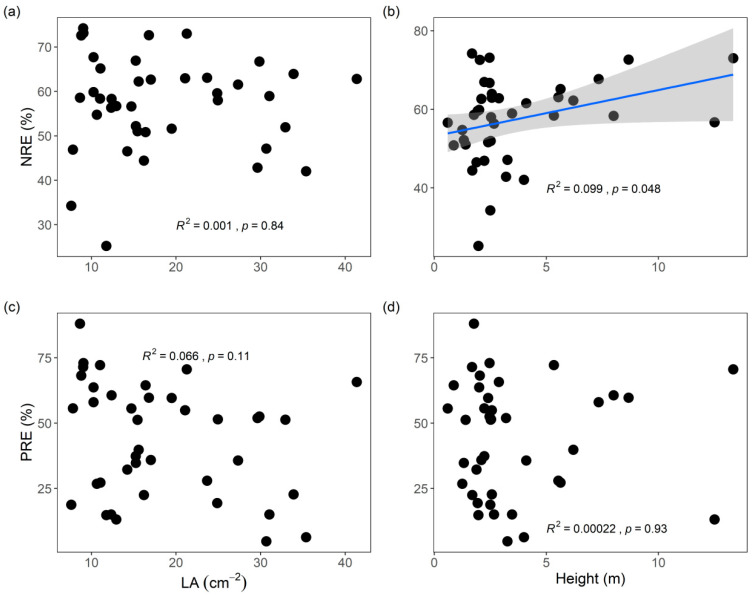
(**a**–**d**) Relationships between leaf resorption efficiency and size traits. Shaded areas indicate confidence intervals. The *R*^2^ and *p* values are reported. Trait abbreviations are the same as those in Table A1.

Our results revealed that the leaf NuRE was significantly correlated with leaf economic traits (*p* < 0.05, Table 2). Notably, NRE and PRE were positively related to N_gr_ (*r* = 0.55 for NRE and 0.31 for PRE, *p* < 0.05; Figure 1a,e) and P_gr_ (*r* = 0.35 for NRE and 0.49 for PRE, *p* < 0.05; Figure 1b,f). Conversely, NRE and PRE were negatively correlated with LDMC (*r* = −0.43 for NRE and −0.41 for PRE, *p* < 0.05; Figure 1c,g) and LMA (*r* = −0.59 for NRE and −0.39 for PRE, *p* < 0.05; Figure 1d,h). In contrast, no significant correlations were detected between the NuRE and size traits (*p* > 0.05), with the exception of a positive relationship between the NRE and height (*r* = 0.32, *p* < 0.05; Table 2 and Figure A2).

**Figure 2:**
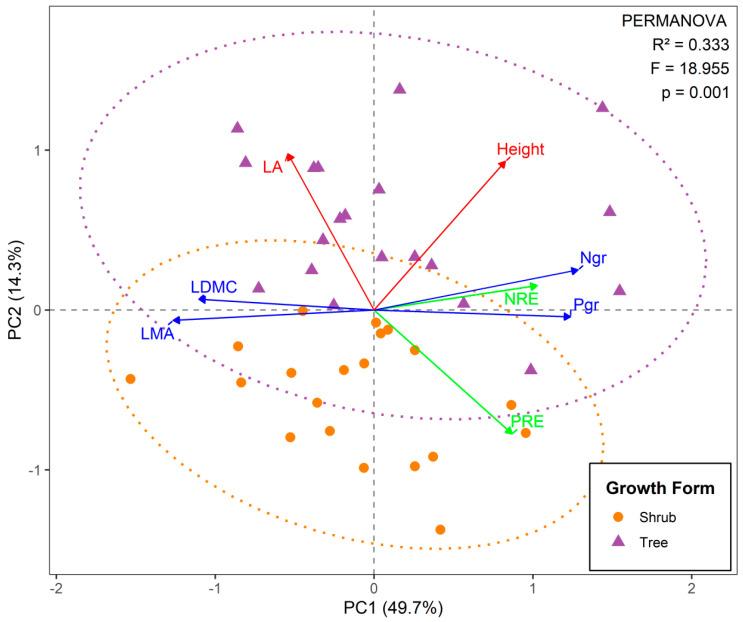
Principal component analysis (PCA) of eight traits among all species in tropical mangrove forests. The three resulting groups are as follows: (1) size-related traits (red) consisting of LA and height; (2) leaf economic traits (blue) comprising N_gr_, P_gr_, LMA, and LDMC; and (3) leaf nutrient resorption (green) consisting of NRE and PRE. Trait abbreviations are the same as those in Table 1.

Principal component analysis (PCA) revealed two dominant ecological strategies: PC1 (49.7% variance) represented a continuum from acquisitive species (high nutrient concentrations [N_gr_ and P_gr_] and resorption efficiency [NRE and PRE]) to conservative species (high LMA and LDMC), whereas PC2 (14.3% variance) primarily reflected size-related variation, with taller plants and larger leaves associated with competitive light acquisition (Figure 2). These findings demonstrate fundamental trade-offs in resource-use strategies among mangrove species.

**Figure A3:**
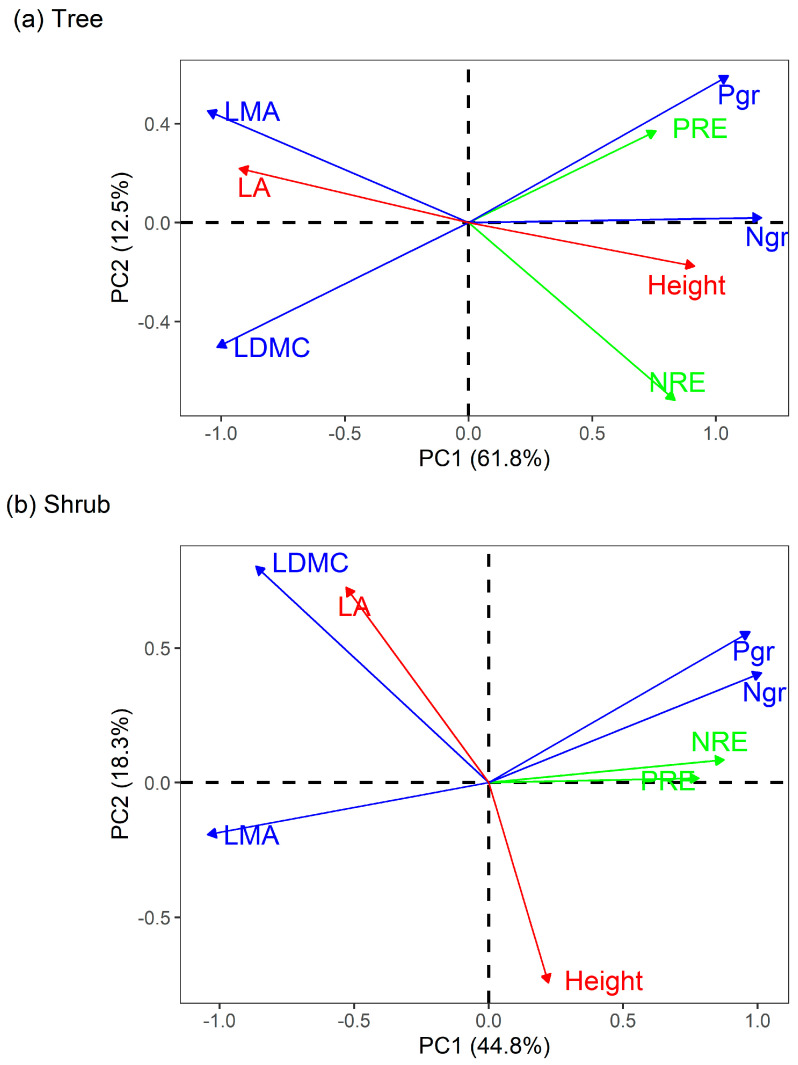
Principal component analysis (PCA) of eight traits between shrubs (**a**) and trees (**b**) in tropical mangrove forests. The three resulting groups are as follows: (1) size-related traits (red) consisting of LA (cm^2^, leaf area) and height (m, plant height); (2) leaf economic traits (blue) comprising N_gr_ (g kg^−1^, green leaf nitrogen content), P_gr_ (g kg^−1^, green leaf phosphorus content), LMA (g m^−2^, leaf mass per area), and LDMC (%, leaf dry mass content); and (3) leaf nutrient resorption (green) consisting of NRE (%, leaf nitrogen resorption efficiency) and PRE (%, leaf phosphorus resorption efficiency).

Shrubs and trees were significantly different within the multivariate trait space (PERMANOVA: *R*^2^ = 0.33, *p* = 0.001; Figure 2). While showing marked divergence in size-related traits (PC2, *p* < 0.001), both growth forms maintained similar economic trade-offs (PC1, *p* > 0.05). These findings suggest that the relationships among leaf traits reflect fundamental constraints that operate independently of the growth form (Figure A3).

**Figure 3:**
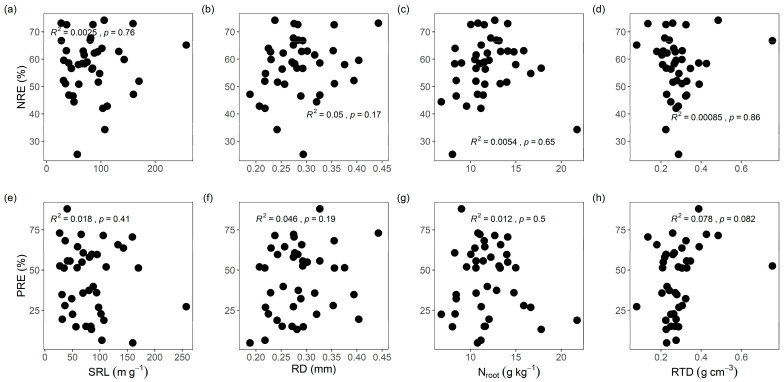
(**a**–**h**) Relationships between leaf nitrogen and phosphorus resorption efficiencies and root economic traits. Shaded areas indicate confidence intervals. The *R*_2_ and *p* values are reported. Trait abbreviations are the same as those in Table 1.

The leaf NRE and PRE did not significantly correlate with root economic traits (SRL, RD, N_root_, and RTD) among the mangrove species (Table 3 and Figure 3).

## 4. Discussion

### 4.1. NRE and PRE Are Mediated by Leaf Economic Traits

Our findings challenge our initial hypothesis by revealing that species with a low structural investment, as indicated by low LMA and LDMC, tend to exhibit high NRE and PRE. This inverse relationship can be explained through the lens of resource allocation trade-offs inherent to the leaf economic spectrum (LES). Similarly, previous studies [3,49] have demonstrated that high-LMA leaves often contain immobile N pools (e.g., structural proteins and secondary compounds such as lignin and phenolics) [50], which reduce resorption efficiency by binding nutrients in recalcitrant tissues. This aligns with the “growth–persistence” trade-off framework, where high NRE/PRE facilitate rapid nutrient cycling for growth [3,51], whereas high LMA/LDMC reflect a conservative strategy prioritizing leaf longevity [49,52]. The observed negative correlations support the classical paradigm that trade-offs exist between resorption efficiency and leaf longevity-related traits [18,53,54]. However, these relationships were inferred indirectly using LMA and LDMC as proxy traits for leaf longevity rather than direct measurements of leaf lifespan. A mechanistic explanation may involve source–sink dynamics: low-LMA leaves likely maintain a stronger phloem transport capacity, enabling efficient nutrient remobilization, whereas structural investments in high-LMA leaves could physically impede hydrolysis or the vascular retrieval of nutrients [55]. While our study did not measure sap flow or phloem/xylem nutrient fluxes, these processes likely mediate the observed resorption efficiencies, particularly for mobile elements. Future studies incorporating hydraulic measurements (e.g., sap flow rates or isotopic tracing) could clarify how source–sink relationships and vascular transport interact with leaf economic traits to shape nutrient resorption strategies.

The positive correlation between NRE/PRE and green leaf nutrients (N_gr_ and P_gr_) presents an apparent paradox, as it contrasts with the “nutrient limitation hypothesis”, which predicts greater resorption in nutrient-poor leaves [2,19,56]. However, this discrepancy may reflect context-dependent adaptations. Mangroves, which thrive in nutrient-limited sediments [30,57], may prioritize the efficient recycling of acquired nutrients to sustain metabolic activity, explaining why N-rich species present a relatively high NRE [49]. These findings suggest that nutrient resorption strategies are not driven solely by leaf-level stoichiometry but rather are modulated by whole-plant nutrient demands and environmental constraints. While some studies report no NuRE–nutrient relationships [8,54,58], our findings align with systems where internal cycling compensates for soil nutrient scarcity [59,60,61]. Thus, the observed pattern likely represents an adaptive strategy to decouple growth from external nutrient availability, a critical advantage in oligotrophic habitats.

**Figure 4:**
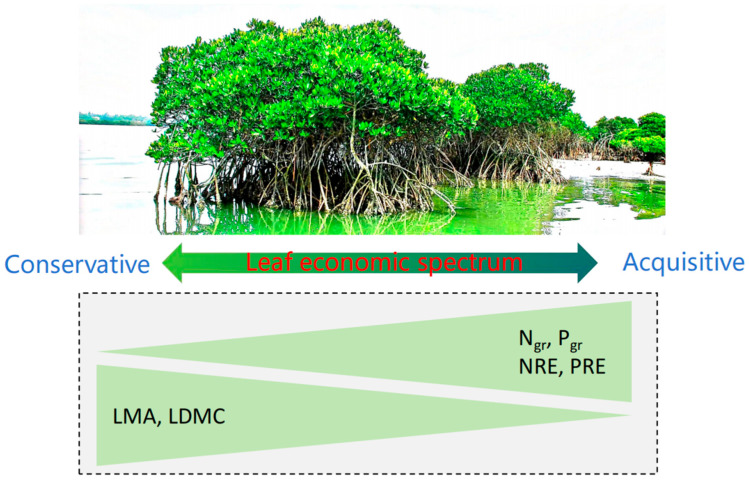
The associations between leaf nutrient resorption efficiency and the leaf economic spectrum. Species with acquisitive traits (high N_gr_ and P_gr_) have greater NRE and PRE values than do species with a conservative strategy (high LMA and LDMC). Trait abbreviations are the same as those in Table 1.

Growth form invariance in NuRE–trait relationships underscores the universality of LES principles across plant functional types (Figure 4). The consistency between trees and shrubs implies that nutrient resorption is governed by overarching economic trade-offs rather than growth-form-specific adaptations. Fast-growing species (high N_gr_/P_gr_ and low LMA) leverage efficient NuRE to fuel rapid growth, whereas conservative species (high LMA/LDMC) prioritize persistence, as predicted by LES [5]. This integration highlights how nutrient conservation strategies are embedded within broader trait syndromes shaping plant ecological success.

### 4.2. Root Economic Traits Had No Effect on NRE and PRE

Contrary to our initial hypothesis, root economic traits (SRL, N_root_, RD, and RTD) had no significant influence on foliar nutrient resorption efficiency (NRE and PRE). This apparent decoupling between above- and belowground nutrient strategies challenges conventional assumptions about plant resource allocation trade-offs. The intricate soil environments that roots encounter have been suggested as a reason for the tenuous connection between root foraging traits and leaf resource economics [62,63]. Nonetheless, additional compensatory mechanisms stemming from the roots, such as the rhizosphere’s influence on nutrient mineralization through the action of extracellular enzymes—a strategy known as root mining—may also contribute to the independence of root foraging traits from aboveground nutrient conservation mechanisms [64,65]. For example, Ding et al. (2023) [65] reported that an increase in nutrient resorption from senescing leaves corresponded to increased availability of nutrients to roots via the process of nutrient mining in the rhizosphere. This nutrient advantage gained from root mining could offset, to some extent, the benefits derived from the modification of foraging-related root traits. As a result, this provides greater flexibility for root foraging traits to evolve in response to a variety of environmental pressures, as highlighted by Kramer-Walter et al. (2016) [62] and Weemstra et al. (2016) [63]. Thus, we propose that the diverse nutrient acquisition tactics utilized by roots, alongside intricate external constraints, may also account for the tenuous link observed between root foraging strategies and leaf nutrient conservation processes.

Our root sampling methodology, while following established protocols [42,43], presents several temporal and spatial considerations that may influence trait interpretation. The rainy season sampling captured peak root activity but potentially missed seasonal trait plasticity [66], and our ≤2 mm diameter threshold, although standard, may have excluded functionally important finer roots (<0.5 mm) in some species [44]. These methodological constraints reflect common trade-offs in root ecology studies between standardization and capturing natural variability, particularly in diverse ecosystems where root systems exhibit high functional plasticity.

### 4.3. Limitations and Future Directions in Root Trait–Resorption Studies

While our study focused on conventional root morphological traits (SRL, N_root_, RD, RTD), we acknowledge that these traits may not fully capture the multidimensional nature of root nutrient acquisition strategies. Recent evidence suggests that physiological traits, such as root exudation rates [67] and symbiotic associations [68], may be more directly linked to nutrient resorption processes. The absence of these functional traits in our analysis could explain why we observed no significant relationships with resorption efficiency. Our trait selection prioritized standardized, measurable characteristics that facilitate cross-study comparisons [24,69]. However, future investigations would benefit from incorporating a broader suite of root functional traits, especially those reflecting biochemical activity and microbial interactions, to better understand belowground nutrient economy.

The lack of observed root trait–resorption relationships may also reflect overriding environmental controls. Soil nutrient heterogeneity, such as localized nutrient hotspots, can disrupt trait–function linkages [70]. Episodic resource pulses from these hotspots or seasonal hydrological shifts may temporarily favor passive uptake or non-trait-mediated pathways, thereby obscuring intrinsic trait effects [71]. These observations highlight the context-dependent nature of root trait ecology, where environmental factors can modulate or override intrinsic trait effects on plant nutrient dynamics.

Furthermore, functional redundancy within root systems allows multiple trait combinations to achieve comparable nutrient uptake efficiencies via distinct pathways. For example, thick mycorrhizal roots can match phosphorus acquisition typically associated with thin, exudative roots [72], whereas slow root turnover can compensate for a lower specific root length (SRL) through prolonged soil exploration [63]. This redundancy emerges from three key mechanisms: (1) biochemical pathways, such as organic acid exudation, increase nutrient solubilization independently of morphology [73]; (2) mycorrhizal networks—functionally extending root absorptive surfaces [74]; and (3) temporal strategies—long-lived roots offset construction costs via sustained nutrient uptake [44]. This multidimensional optimization explains why no single root morphological trait predicted resorption patterns in our study, as plants employ context-dependent solutions: different trait combinations yield similar functional outcomes under varying environmental conditions.

### 4.4. Integrating Above- and Belowground Strategies for Sustainable Mangrove Management

On the basis of our findings and methodological constraints, we propose the following evidence-based recommendations for mangrove restoration and management:

- In nitrogen-deficient soils, *Sonneratia caseolaris* is recommended because of its high NRE (65.60%) and low LMA (103.33 g m^−2^). Its trait profile is especially advantageous in tidal areas where sediment nitrogen availability is naturally reduced.
- For phosphorus-limited sites, *Lumnitzera racemosa* is an optimal choice given its high PRE (71.24%), thick-rooted morphology (RD = 0.36 mm), and reliance on mycorrhizal symbiosis, which are traits that are well suited for nutrient acquisition in compacted sediments.

However, our single-season sampling may not fully capture phenotypic plasticity; for example, preliminary data for *Rhizophora stylosa* indicate a 21.5% variation in NRE across seasons [23]. Therefore, we recommend multi-year monitoring to assess trait stability before large-scale restoration.

This study provides valuable insights into mangrove nutrient resorption strategies, but certain limitations must be acknowledged. For example, unmeasured water table fluctuations may influence root nutrient uptake dynamics [75], potentially masking trait–resorption relationships. Future research should incorporate continuous hydrological monitoring (e.g., piezometers) to disentangle waterlogging effects. Additionally, sampling during peak abscission might overlook early or late leaf senescence stages [76], potentially biasing resorption efficiency estimates. Employing litterfall traps to track full leaf lifespans could improve accuracy. Further studies should include trait–environment experiments (e.g., gradients of nutrients and salinity stress), high-resolution root imaging techniques (such as X-ray tomography), and isotopic tracers (e.g., ^15^N labeling) to better elucidate the drivers of resorption plasticity and nutrient conservation mechanisms in mangroves.

## 5. Conclusions

This study revealed that mangroves have evolved a unique nutrient conservation strategy in which leaf economic traits, such as low LMA and LDMC, increase NRE and PRE independently of root nutrient acquisition traits. This decoupling represents a fundamental departure from the integrated whole-plant strategies typically observed in terrestrial ecosystems. This adaptation likely reflects a specialized response to the nutrient-poor coastal environments mangroves inhabit, challenging conventional paradigms of plant economics, which often assume coordinated trait syndromes.

Our findings suggest that mangroves achieve resilience and optimize nutrient conservation through modular responses to multiple environmental stressors. Consequently, restoration efforts should consider targeting aboveground traits related to nutrient preservation independent of belowground traits associated with stress tolerance. This modular approach could improve the success of mangrove restoration programs, especially in challenging coastal conditions where resource limitations are prevalent.

## Figures and Tables

**Table 1:** Leaf nutrient resorption efficiency, size traits, and economic traits (units) and their mean values, ranges, and coefficients of variation (CVs) in this study.

Variables	Abbreviation	Mean	Min	Max	CV (%)
Leaf nitrogen resorption efficiency (%)	NRE	57.37	25.24	74.23	18.74
Leaf phosphorus resorption efficiency (%)	PRE	43.79	4.80	88.05	49.59
Green leaf nitrogen content (g kg^−1^)	N_gr_	20.37	9.12	35.25	29.59
Green leaf phosphorus content (g kg^−1^)	P_gr_	1.29	0.60	3.20	43.25
Leaf mass per area (g m^−2^)	LMA	159.39	89.26	242.71	22.84
Leaf dry mass content (%)	LDMC	25.38	14.42	34.34	16.58
Leaf area (cm^2^)	LA	18.44	7.62	41.36	48.84
Plant height (m)	Height	3.58	0.60	13.33	80.62
Specific root length (m g^−1^)	SRL	82.80	26.69	257.19	57.14
Root diameter (mm)	RD	0.29	0.19	0.44	20.01
Root nitrogen content (g kg^−1^)	N_root_	11.92	6.77	21.73	24.41
Root tissue density (g cm^−3^)	RTD	0.28	0.08	0.75	37.14

**Table 2:** Pearson’s correlation analyses among leaf nutrient resorption efficiency, size traits, and leaf economic traits for all species. Correlation coefficients are given to the lower left of the diagonal. Bold indicates a significant correlation. Trait abbreviations are the same as those in Table 1. * *p* < 0.05. ** *p* < 0.01. *** *p* < 0.001.

	NRE	PRE	N_gr_	P_gr_	LDMC	LMA	LA
PRE	0.48 **						
N_gr_	0.55 ***	0.31 *					
P_gr_	0.35 *	0.49 **	0.80 ***				
LDMC	−0.43 **	−0.41 **	−0.51 ***	−0.55 ***			
LMA	−0.59 ***	−0.39 *	−0.72 ***	−0.63 ***	0.51 ***		
LA	−0.03	−0.26	−0.23	−0.26	0.29	0.32 *	
Height	0.32 *	0.01	−0.01	0.40 *	−0.42 **	−0.44 **	−0.01

**Table 3:** Pearson’s correlation analyses between leaf nutrient resorption efficiency and root economic traits for all species. Correlation coefficients are given to the lower left of the diagonal. Bold indicates a significant correlation. Trait abbreviations are the same as those in Table 1. ** *p* < 0.01. *** *p* < 0.001.

	NRE	PRE	SRL	RD	N_root_
PRE	0.48 **				
SRL	0.05	−0.13			
RD	0.22	0.21	−0.63 ***		
N_root_	0.07	−0.11	0.10	−0.09	
RTD	0.03	0.28	−0.45 **	−0.004	−0.09

## Data Availability

The data that support the findings of this study are available from the corresponding author upon reasonable request. The data are not publicly available due to privacy restrictions.

## Abbreviations

The following abbreviations are used in this manuscript:

NRE	Nitrogen resorption efficiency
PRE	Phosphorus resorption efficiency
N_gr_	Green leaf nitrogen content
P_gr_	Green leaf phosphorus content
LMA	Leaf mass per area
LDMC	Leaf dry mass content
LA	Leaf area
Height	Plant height
SRL	Specific root length
RD	Root diameter
N_root_	Root nitrogen content
RTD	Root tissue density

## Appendix A

**Table A1:** Lists of species, growth forms, and plant traits (means ± SEs) used in this study.

Species	Growth Forms	NRE	PRE	N_gr_	P_gr_	LMA	LDMC	LA	Height	SRL	RD	N_root_	RTD
*Avicennia marina*	Shrub	55.79 ± 8.27	45.21 ± 13.12	29.96 ± 2.57	1.86 ± 0.41	133.37 ± 2.01	24.38 ± 0.63	9.38 ± 1.36	1.86 ± 0.26	113.43 ± 20.09	0.23 ± 0.01	15.38 ± 4.94	0.33 ± 0.11
*Kandelia obovata*	Shrub	62.24 ± 2.19	35.83 ± 8.15	20.39 ± 1.11	1.24 ± 0.27	161.38 ± 12.23	27.01 ± 1.03	16.41 ± 3.66	2.39 ± 0.13	81.31 ± 10.96	0.27 ± 0.03	13.34 ± 1.39	0.24 ± 0.02
*Aegiceras corniculatum*	Shrub	52.54 ± 1.37	57.77 ± 2.82	16.25 ± 0.20	1.15 ± 0.14	158.31 ± 18.76	30.66 ± 1.06	16.51 ± 2.10	1.32 ± 0.40	58.68 ± 26.29	0.29 ± 0.05	12.35 ± 1.55	0.33 ± 0.06
*Sonneratia caseolaris*	Tree	65.60 ± 4.18	65.81 ± 3.25	30.70 ± 1.13	2.50 ± 0.48	103.33 ± 17.92	18.62 ± 2.99	15.36 ± 4.64	8.83 ± 1.66	94.96 ± 43.58	0.28 ± 0.01	10.85 ± 2.45	0.26 ± 0.12
*Sonneratia apetala*	Tree	62.96 ± 2.37	34.56 ± 9.52	22.65 ± 0.58	1.18 ± 0.28	140.43 ± 9.07	24.30 ± 1.47	12.46 ± 2.37	7.92 ± 1.57	127.66 ± 86.40	0.27 ± 0.01	13.25 ± 3.05	0.19 ± 0.07
*Ceriops tagal*	Shrub	42.10 ± 5.86	26.09 ± 4.59	12.47 ± 1.28	0.80 ± 0.23	213.57 ± 22.69	29.66 ± 3.54	14.36 ± 1.91	1.72 ± 0.14	46.60 ± 10.86	0.32 ± 0.05	7.90 ± 0.78	0.28 ± 0.03
*Lumnitzera racemosa*	Shrub	62.82 ± 6.28	71.24 ± 6.69	16.72 ± 1.02	1.02 ± 0.34	159.30 ± 7.04	18.75 ± 0.24	8.57 ± 0.54	2.13 ± 0.15	36.23 ± 6.62	0.36 ± 0.05	10.67 ± 1.16	0.32 ± 0.05
*Rhizophora stylosa*	Tree	46.00 ± 2.28	28.62 ± 13.3	16.81 ± 0.59	1.09 ± 0.35	219.07 ± 7.66	26.30 ± 0.99	32.15 ± 2.56	3.25 ± 0.30	136.25 ± 33.58	0.21 ± 0.01	10.51 ± 0.68	0.28 ± 0.04
*Bruguiera sexangula*	Tree	61.87 ± 1.94	37.87 ± 8.35	18.80 ± 0.70	1.01 ± 0.16	132.00 ± 13.28	28.03 ± 0.87	25.84 ± 2.74	3.12 ± 0.82	38.32 ± 13.98	0.36 ± 0.05	14.00 ± 1.74	0.39 ± 0.25
*Bruguiera gymnorrhiza*	Tree	61.82 ± 1.07	34.84 ± 11.16	18.96 ± 0.68	1.03 ± 0.20	173.13 ± 5.75	26.13 ± 1.84	33.41 ± 5.95	3.25 ± 0.34	94.6 ± 29.28	0.28 ± 0.04	10.93 ± 2.09	0.23 ± 0.04

**Table A2:** Phylogenetic signals of plant traits based on Blomberg’s K and Pagel’s λ values. A K or λ close to 0 indicates a lack of phylogenetic conservatism, whereas a K or λ close to 1 indicates a strong phylogenetic signal [47,48]. Trait abbreviations are the same as those in Table A1.

	K	P	λ	P
NRE	0.08	0.36	<0.0001	1.00
PRE	0.10	0.44	0.25	0.48
N_gr_	0.04	0.48	0.53	0.38
P_gr_	0.02	0.64	0.58	0.20
LMA	0.30	0.16	0.09	0.81
LDMC	0.03	0.60	0.24	0.64
LA	0.44	0.05	0.37	0.35
Height	0.03	0.64	0.73	0.07
SRL	0.12	0.19	<0.0001	1.00
RD	0.28	0.08	<0.0001	1.00
N_root_	0.07	0.36	<0.0001	1.00
RTD	0.01	0.76	0.20	0.67
